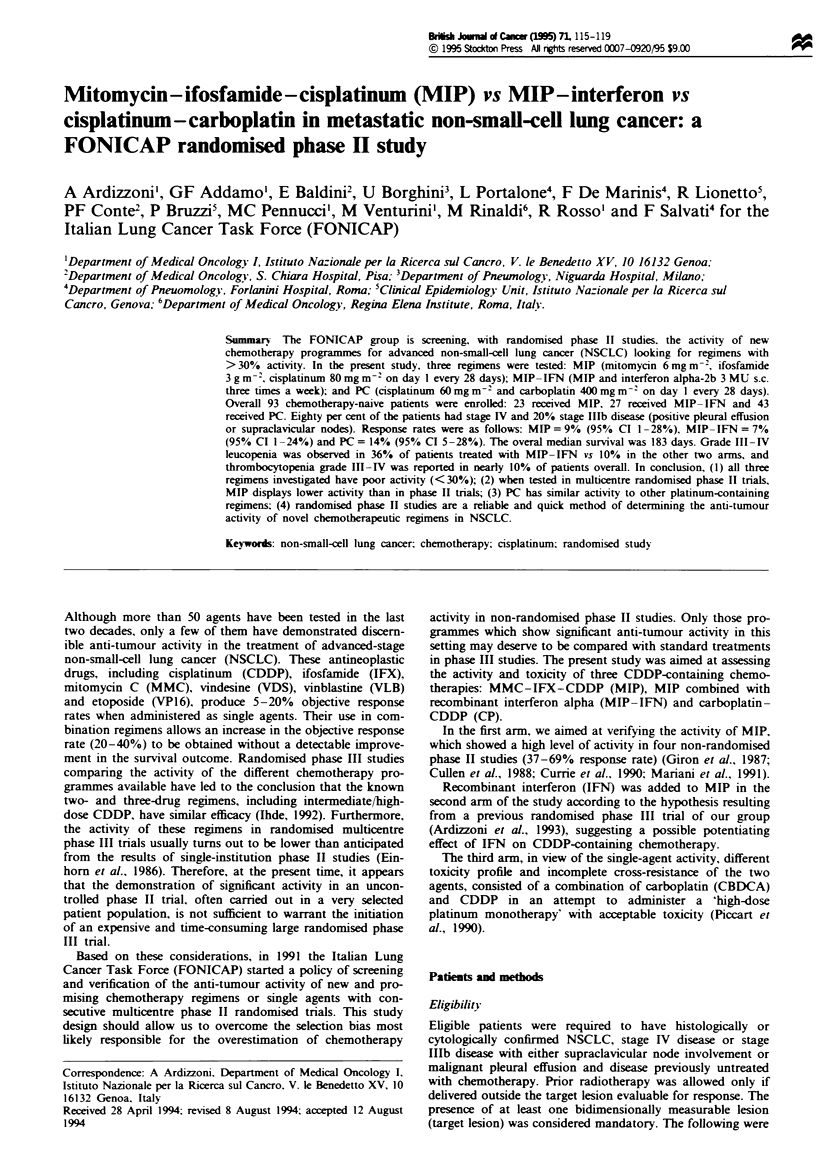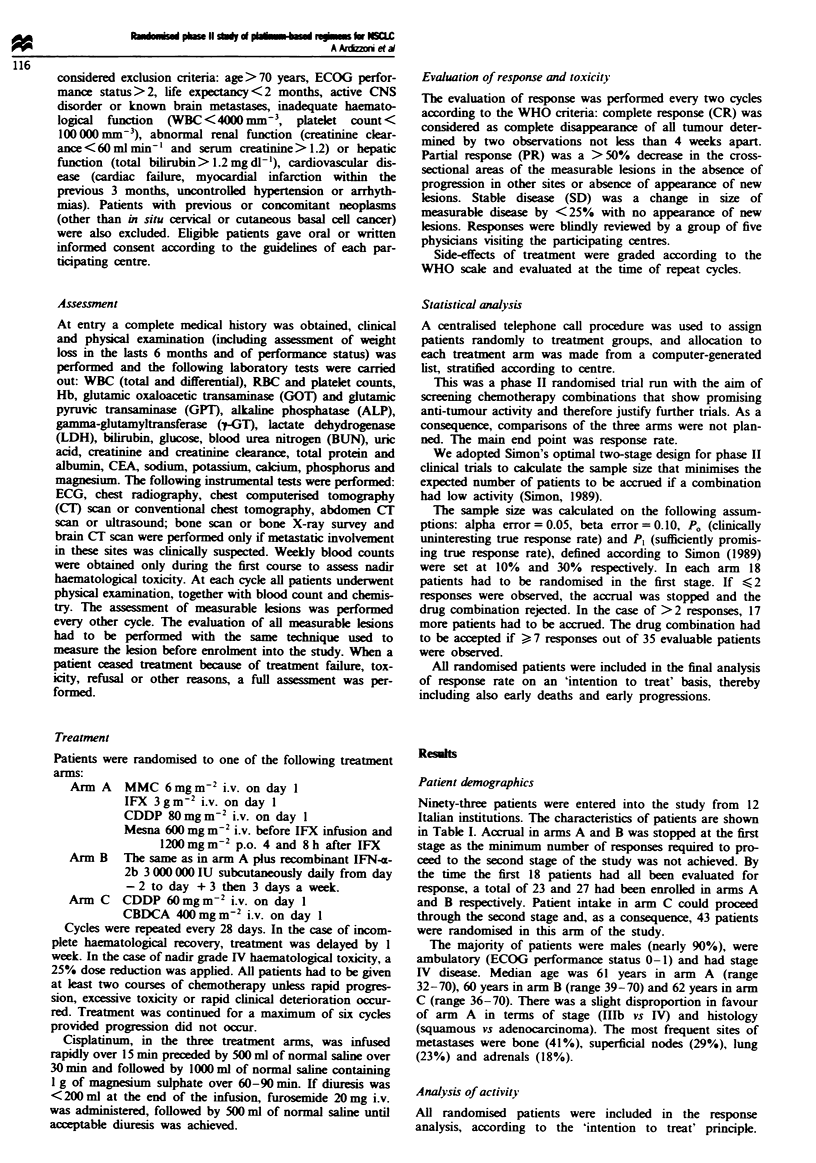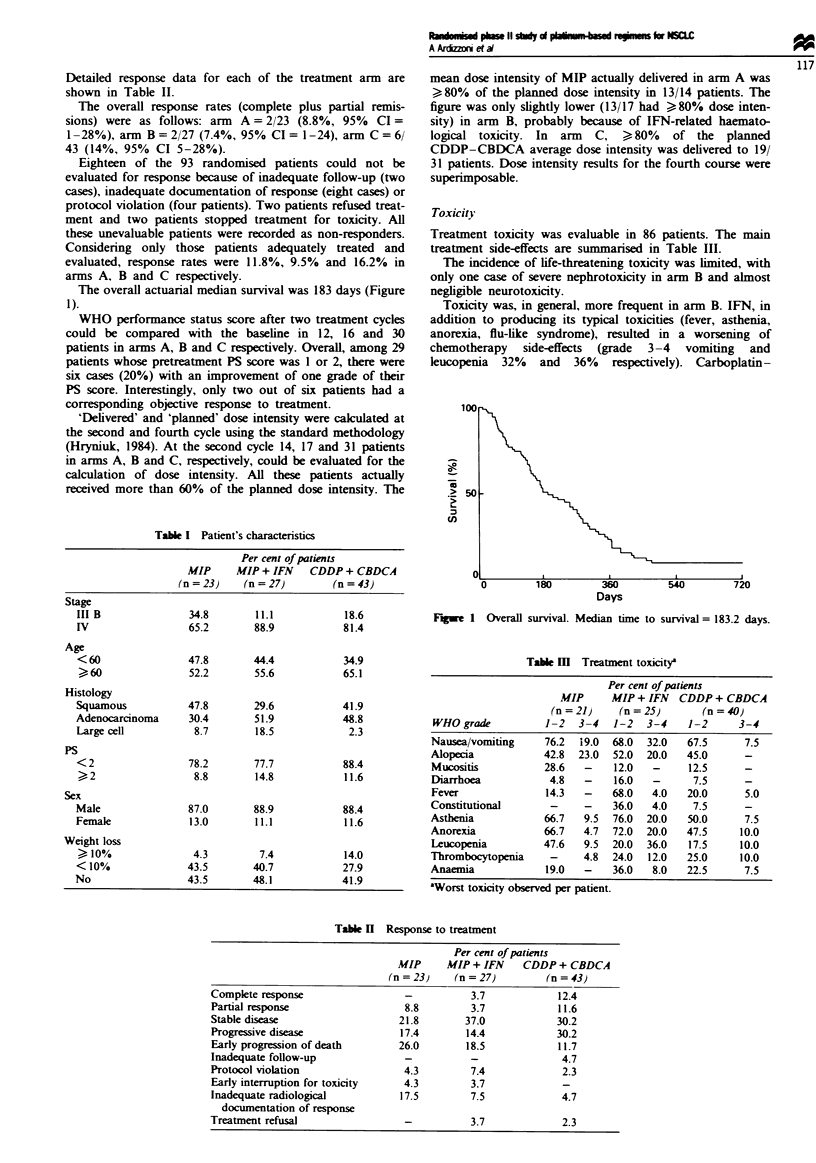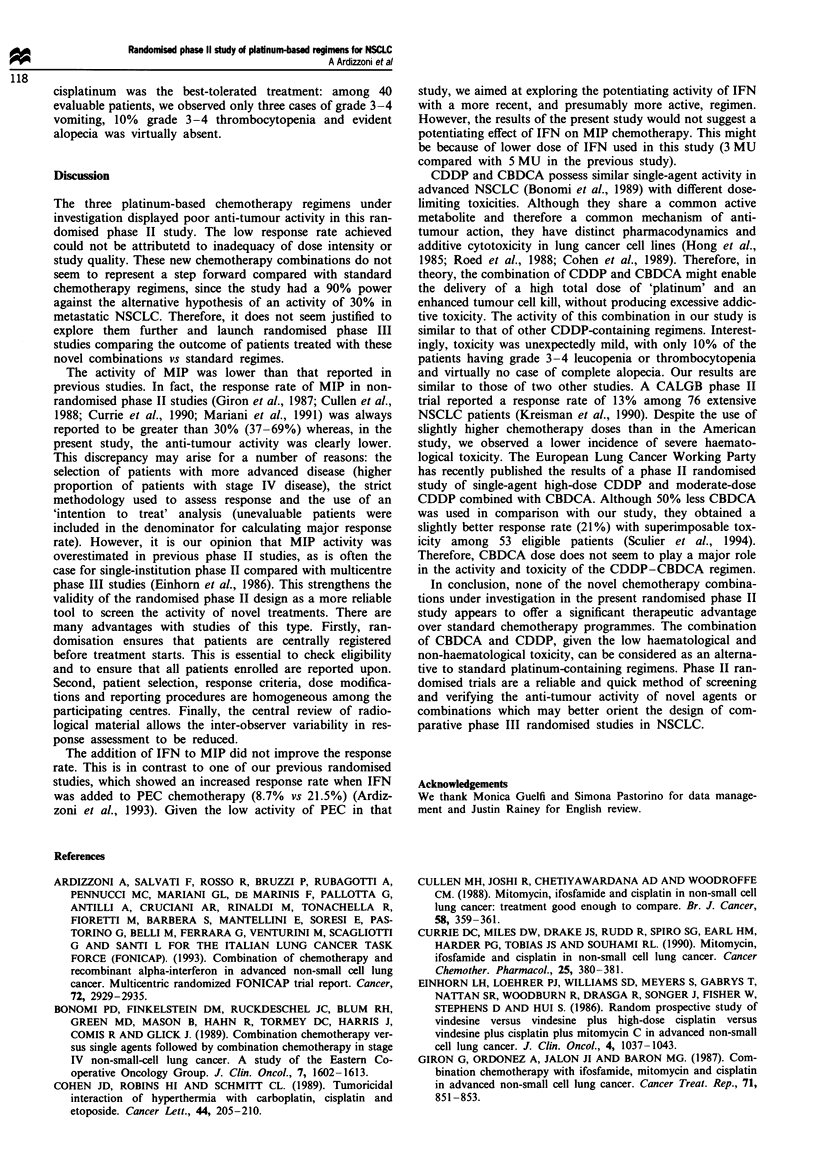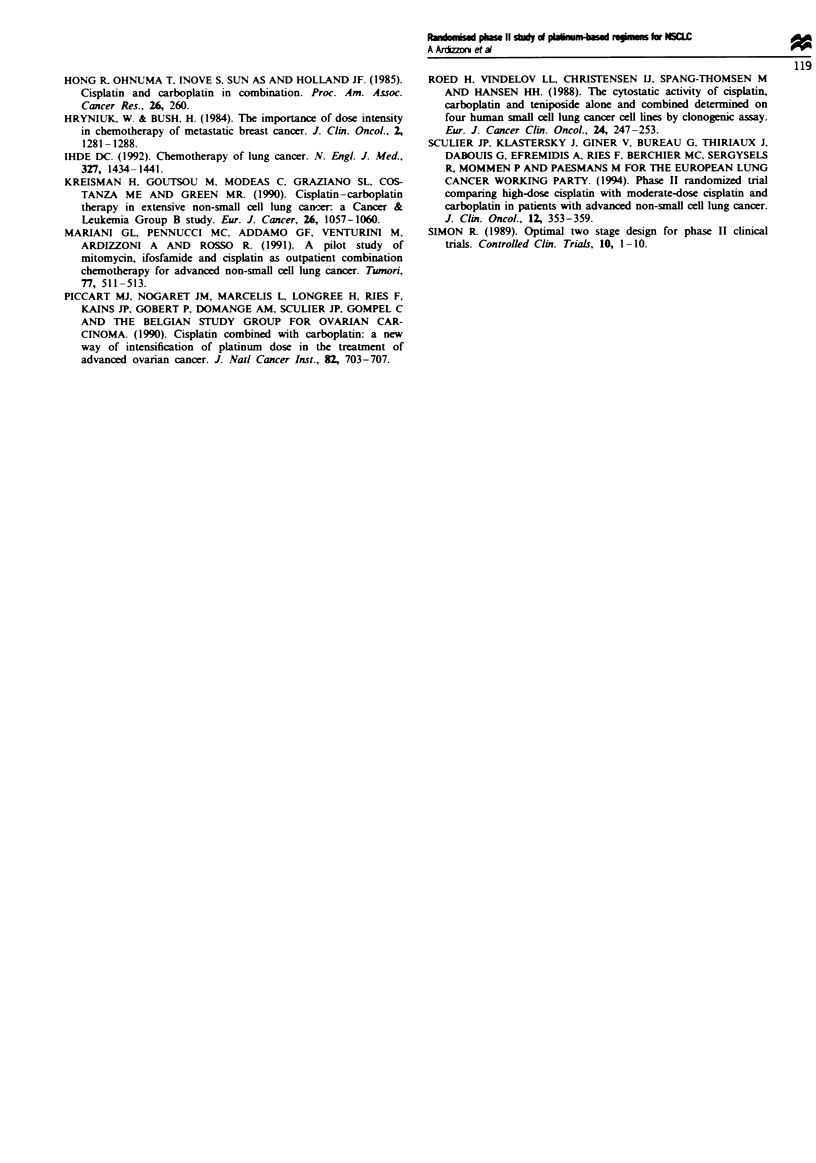# Mitomycin-ifosfamide-cisplatinum (MIP) vs MIP-interferon vs cisplatinum-carboplatin in metastatic non-small-cell lung cancer: a FONICAP randomised phase II study. Italian Lung Cancer Task Force.

**DOI:** 10.1038/bjc.1995.23

**Published:** 1995-01

**Authors:** A. Ardizzoni, G. F. Addamo, E. Baldini, U. Borghini, L. Portalone, F. De Marinis, R. Lionetto, P. F. Conte, P. Bruzzi, M. C. Pennucci

**Affiliations:** Department of Medical Oncology I, Istituto Nazionale per la Ricerca sul Cancro, V. le Benedetto XY, Genoa, Italy.

## Abstract

The FONICAP group is screening, with randomised phase II studies, the activity of new chemotherapy programmes for advanced non-small-cell lung cancer (NSCLC) looking for regimens with > 30% activity. In the present study, three regimens were tested: MIP (mitomycin 6 mg m-2, ifosfamide 3 g m-2, cisplatinum 80 mg m-2 on day 1 every 28 days); MIP-IFN (MIP and interferon alpha-2b 3 MU s.c. three times a week); and PC (cisplatinum 60 mg m-2 and carboplatin 400 mg m-2 on day 1 every 28 days). Overall 93 chemotherapy-naive patients were enrolled: 23 received MIP, 27 received MIP-IFN and 43 received PC. Eighty per cent of the patients had stage IV and 20% stage IIIb disease (positive pleural effusion or supraclavicular nodes). Response rates were as follows: MIP = 9% (95% CI 1-28%), MIP-IFN = 7% (95% CI 1-24%) and PC = 14% (95% CI 5-28%). The overall median survival was 183 days. Grade III-IV leucopenia was observed in 36% of patients treated with MIP-IFN vs 10% in the other two arms, and thrombocytopenia grade III-IV was reported in nearly 10% of patients overall. In conclusion, (1) all three regimens investigated have poor activity (< 30%); (2) when tested in multicentre randomised phase II trials, MIP displays lower activity than in phase II trials; (3) PC has similar activity to other platinum-containing regimens; (4) randomised phase II studies are a reliable and quick method of determining the anti-tumour activity of novel chemotherapeutic regimens in NSCLC.


British Journal of Cancer (1995) 71, 115-119

© 1995 Stockton Press All rights reserved 0007-0920/95 $9.00

### Mitomycin–ifosfamide–cisplatinum (MIP) *vs* MIP–interferon *vs* cisplatinum–carboplatin in metastatic non-small-cell lung cancer: a FONICAP randomised phase II study

A Ardizzoni[1], GF Addamo[1], E Baldini[2], U Borghini[3], L Portalone[4], F De Marinis[4], R Lionetto[5], PF Conte[2], P Bruzzi[5], MC Pennucci[1], M Venturini[1], M Rinaldi[6], R Rosso[1] and F Salvati[4] for the Italian Lung Cancer Task Force (FONICAP)

[1]*Department of Medical Oncology I, Istituto Nazionale per la Ricerca sul Cancro, V. le Benedetto XV, 10 16132 Genoa;* [2]*Department of Medical Oncology, S. Chiara Hospital, Pisa;* [3]*Department of Pneumology, Niguarda Hospital, Milano;* [4]*Department of Pneuomology, Forlanini Hospital, Roma;* [5]*Clinical Epidemiology Unit, Istituto Nazionale per la Ricerca sul Cancro, Genova;* [6]*Department of Medical Oncology, Regina Elena Institute, Roma, Italy.*

**Summary** The FONICAP group is screening, with randomised phase II studies, the activity of new chemotherapy programmes for advanced non-small-cell lung cancer (NSCLC) looking for regimens with >30% activity. In the present study, three regimens were tested: MIP (mitomycin 6 mg m$^{-2}$, ifosfamide 3 g m$^{-2}$, cisplatinum 80 mg m$^{-2}$ on day 1 every 28 days); MIP–IFN (MIP and interferon alpha-2b 3 MU s.c. three times a week); and PC (cisplatinum 60 mg m$^{-2}$ and carboplatin 400 mg m$^{-2}$ on day 1 every 28 days). Overall 93 chemotherapy-naive patients were enrolled: 23 received MIP, 27 received MIP–IFN and 43 received PC. Eighty per cent of the patients had stage IV and 20% stage IIIb disease (positive pleural effusion or supraclavicular nodes). Response rates were as follows: MIP = 9% (95% CI 1–28%), MIP–IFN = 7% (95% CI 1–24%) and PC = 14% (95% CI 5–28%). The overal median survival was 183 days. Grade III–IV leucopenia was observed in 36% of patients treated with MIP–IFN *vs* 10% in the other two arms, and thrombocytopenia grade III–IV was reported in nearly 10% of patients overall. In conclusion, (1) all three regimens investigated have poor activity (<30%); (2) when tested in multicentre randomised phase II trials, MIP displays lower activity than in phase II trials; (3) PC has similar activity to other platinum-containing regimens; (4) randomised phase II studies are a reliable and quick method of determining the anti-tumour activity of novel chemotherapeutic regimens in NSCLC.

**Keywords**: non-small-cell lung cancer; chemotherapy; cisplatinum; randomised study

Although more than 50 agents have been tested in the last two decades, only a few of them have demonstrated discernible anti-tumour activity in the treatment of advanced-stage non-small-cell lung cancer (NSCLC). These antineoplastic drugs, including cisplatinum (CDDP), ifosfamide (IFX), mitomycin C (MMC), vindesine (VDS), vinblastine (VLB) and etoposide (VP16), produce 5–20% objective response rates when administered as single agents. Their use in combination regimens allows an increase in the objective response rate (20–40%) to be obtained without a detectable improvement in the survival outcome. Randomised phase III studies comparing the activity of the different chemotherapy programmes available have led to the conclusion that the known two- and three-drug regimens, including intermediate/high-dose CDDP, have similar efficacy (Ihde, 1992). Furthermore, the activity of these regimens in randomised multicentre phase III trials usually turns out to be lower than anticipated from the results of single-institution phase II studies (Einhorn *et al.*, 1986). Therefore, at the present time, it appears that the demonstration of significant activity in an uncontrolled phase II trial, often carried out in a very selected patient population, is not sufficient to warrant the initiation of an expensive and time-consuming large randomised phase III trial.

Based on these considerations, in 1991 the Italian Lung Cancer Task Force (FONICAP) started a policy of screening and verification of the anti-tumour activity of new and promising chemotherapy regimens or single agents with consecutive multicentre phase II randomised trials. This study design should allow us to overcome the selection bias most likely responsible for the overestimation of chemotherapy activity in non-randomised phase II studies. Only those programmes which show significant anti-tumour activity in this setting may deserve to be compared with standard treatments in phase III studies. The present study was aimed at assessing the activity and toxicity of three CDDP-containing chemotherapies: MMC–IFX–CDDP (MIP), MIP combined with recombinant interferon alpha (MIP–IFN) and carboplatin–CDDP (CP).

In the first arm, we aimed at verifying the activity of MIP, which showed a high level of activity in four non-randomised phase II studies (37–69% response rate) (Giron *et al.*, 1987; Cullen *et al.*, 1988; Currie *et al.*, 1990; Mariani *et al.*, 1991).

Recombinant interferon (IFN) was added to MIP in the second arm of the study according to the hypothesis resulting from a previous randomised phase III trial of our group (Ardizzoni *et al.*, 1993), suggesting a possible potentiating effect of IFN on CDDP-containing chemotherapy.

The third arm, in view of the single-agent activity, different toxicity profile and incomplete cross-resistance of the two agents, consisted of a combination of carboplatin (CBDCA) and CDDP in an attempt to administer a 'high-dose platinum monotherapy' with acceptable toxicity (Piccart *et al.*, 1990).

### Patients and methods

### *Eligibility*

Eligible patients were required to have histologically or cytologically confirmed NSCLC, stage IV disease or stage IIIb disease with either supraclavicular node involvement or malignant pleural effusion and disease previously untreated with chemotherapy. Prior radiotherapy was allowed only if delivered outside the target lesion evaluable for response. The presence of at least one bidimensionally measurable lesion (target lesion) was considered mandatory. The following were

Correspondence: A Ardizzoni, Department of Medical Oncology I, Istituto Nazionale per la Ricerca sul Cancro, V. le Benedetto XV, 10 16132 Genoa, Italy
Received 28 April 1994; revised 8 August 1994; accepted 12 August 1994

considered exclusion criteria: age > 70 years, ECOG performance status > 2, life expectancy < 2 months, active CNS disorder or known brain metastases, inadequate haematological function (WBC < 4000 $mm^{-3}$, platelet count < 100 000 $mm^{-3}$), abnormal renal function (creatinine clearance < 60 ml $min^{-1}$ and serum creatinine > 1.2) or hepatic function (total bilirubin > 1.2 mg $dl^{-1}$), cardiovascular disease (cardiac failure, myocardial infarction within the previous 3 months, uncontrolled hypertension or arrhythmias). Patients with previous or concomitant neoplasms (other than *in situ* cervical or cutaneous basal cell cancer) were also excluded. Eligible patients gave oral or written informed consent according to the guidelines of each participating centre.

*Assessment*

At entry a complete medical history was obtained, clinical and physical examination (including assessment of weight loss in the lasts 6 months and of performance status) was performed and the following laboratory tests were carried out: WBC (total and differential), RBC and platelet counts, Hb, glutamic oxaloacetic transaminase (GOT) and glutamic pyruvic transaminase (GPT), alkaline phosphatase (ALP), gamma-glutamyltransferase ($\gamma$-GT), lactate dehydrogenase (LDH), bilirubin, glucose, blood urea nitrogen (BUN), uric acid, creatinine and creatinine clearance, total protein and albumin, CEA, sodium, potassium, calcium, phosphorus and magnesium. The following instrumental tests were performed: ECG, chest radiography, chest computerised tomography (CT) scan or conventional chest tomography, abdomen CT scan or ultrasound; bone scan or bone X-ray survey and brain CT scan were performed only if metastatic involvement in these sites was clinically suspected. Weekly blood counts were obtained only during the first course to assess nadir haematological toxicity. At each cycle all patients underwent physical examination, together with blood count and chemistry. The assessment of measurable lesions was performed every other cycle. The evaluation of all measurable lesions had to be performed with the same technique used to measure the lesion before enrolment into the study. When a patient ceased treatment because of treatment failure, toxicity, refusal or other reasons, a full assessment was performed.

*Treatment*

Patients were randomised to one of the following treatment arms:

Arm A MMC 6 mg $m^{-2}$ i.v. on day 1
IFX 3 g $m^{-2}$ i.v. on day 1
CDDP 80 mg $m^{-2}$ i.v. on day 1
Mesna 600 mg $m^{-2}$ i.v. before IFX infusion and 1200 mg $m^{-2}$ p.o. 4 and 8 h after IFX

Arm B The same as in arm A plus recombinant IFN-$\alpha$-2b 3 000 000 IU subcutaneously daily from day − 2 to day + 3 then 3 days a week.

Arm C CDDP 60 mg $m^{-2}$ i.v. on day 1
CBDCA 400 mg $m^{-2}$ i.v. on day 1

Cycles were repeated every 28 days. In the case of incomplete haematological recovery, treatment was delayed by 1 week. In the case of nadir grade IV haematological toxicity, a 25% dose reduction was applied. All patients had to be given at least two courses of chemotherapy unless rapid progression, excessive toxicity or rapid clinical deterioration occurred. Treatment was continued for a maximum of six cycles provided progression did not occur.

Cisplatinum, in the three treatment arms, was infused rapidly over 15 min preceded by 500 ml of normal saline over 30 min and followed by 1000 ml of normal saline containing 1 g of magnesium sulphate over 60–90 min. If diuresis was < 200 ml at the end of the infusion, furosemide 20 mg i.v. was administered, followed by 500 ml of normal saline until acceptable diuresis was achieved.

*Evaluation of response and toxicity*

The evaluation of response was performed every two cycles according to the WHO criteria: complete response (CR) was considered as complete disappearance of all tumour determined by two observations not less than 4 weeks apart. Partial response (PR) was a > 50% decrease in the cross-sectional areas of the measurable lesions in the absence of progression in other sites or absence of appearance of new lesions. Stable disease (SD) was a change in size of measurable disease by < 25% with no appearance of new lesions. Responses were blindly reviewed by a group of five physicians visiting the participating centres.

Side-effects of treatment were graded according to the WHO scale and evaluated at the time of repeat cycles.

*Statistical analysis*

A centralised telephone call procedure was used to assign patients randomly to treatment groups, and allocation to each treatment arm was made from a computer-generated list, stratified according to centre.

This was a phase II randomised trial run with the aim of screening chemotherapy combinations that show promising anti-tumour activity and therefore justify further trials. As a consequence, comparisons of the three arms were not planned. The main end point was response rate.

We adopted Simon's optimal two-stage design for phase II clinical trials to calculate the sample size that minimises the expected number of patients to be accrued if a combination had low activity (Simon, 1989).

The sample size was calculated on the following assumptions: alpha error = 0.05, beta error = 0.10, $P_0$ (clinically uninteresting true response rate) and $P_1$ (sufficiently promising true response rate), defined according to Simon (1989) were set at 10% and 30% respectively. In each arm 18 patients had to be randomised in the first stage. If $\leqslant 2$ responses were observed, the accrual was stopped and the drug combination rejected. In the case of > 2 responses, 17 more patients had to be accrued. The drug combination had to be accepted if $\geqslant 7$ responses out of 35 evaluable patients were observed.

All randomised patients were included in the final analysis of response rate on an 'intention to treat' basis, thereby including also early deaths and early progressions.

### Results

*Patient demographics*

Ninety-three patients were entered into the study from 12 Italian institutions. The characteristics of patients are shown in Table I. Accrual in arms A and B was stopped at the first stage as the minimum number of responses required to proceed to the second stage of the study was not achieved. By the time the first 18 patients had all been evaluated for response, a total of 23 and 27 had been enrolled in arms A and B respectively. Patient intake in arm C could proceed through the second stage and, as a consequence, 43 patients were randomised in this arm of the study.

The majority of patients were males (nearly 90%), were ambulatory (ECOG performance status 0–1) and had stage IV disease. Median age was 61 years in arm A (range 32–70), 60 years in arm B (range 39–70) and 62 years in arm C (range 36–70). There was a slight disproportion in favour of arm A in terms of stage (IIIb *vs* IV) and histology (squamous *vs* adenocarcinoma). The most frequent sites of metastases were bone (41%), superficial nodes (29%), lung (23%) and adrenals (18%).

*Analysis of activity*

All randomised patients were included in the response analysis, according to the 'intention to treat' principle.

Detailed response data for each of the treatment arm are shown in Table II.

The overall response rates (complete plus partial remissions) were as follows: arm A = 2/23 (8.8%, 95% CI = 1-28%), arm B = 2/27 (7.4%, 95% CI = 1-24), arm C = 6/43 (14%, 95% CI 5-28%).

Eighteen of the 93 randomised patients could not be evaluated for response because of inadequate follow-up (two cases), inadequate documentation of response (eight cases) or protocol violation (four patients). Two patients refused treatment and two patients stopped treatment for toxicity. All these unevaluable patients were recorded as non-responders. Considering only those patients adequately treated and evaluated, response rates were 11.8%, 9.5% and 16.2% in arms A, B and C respectively.

The overall actuarial median survival was 183 days (Figure 1).

WHO performance status score after two treatment cycles could be compared with the baseline in 12, 16 and 30 patients in arms A, B and C respectively. Overall, among 29 patients whose pretreatment PS score was 1 or 2, there were six cases (20%) with an improvement of one grade of their PS score. Interestingly, only two out of six patients had a corresponding objective response to treatment.

'Delivered' and 'planned' dose intensity were calculated at the second and fourth cycle using the standard methodology (Hryniuk, 1984). At the second cycle 14, 17 and 31 patients in arms A, B and C, respectively, could be evaluated for the calculation of dose intensity. All these patients actually received more than 60% of the planned dose intensity. The mean dose intensity of MIP actually delivered in arm A was ≥80% of the planned dose intensity in 13/14 patients. The figure was only slightly lower (13/17 had ≥80% dose intensity) in arm B, probably because of IFN-related haematological toxicity. In arm C, ≥80% of the planned CDDP-CBDCA average dose intensity was delivered to 19/31 patients. Dose intensity results for the fourth course were superimposable.

### *Toxicity*

Treatment toxicity was evaluable in 86 patients. The main treatment side-effects are summarised in Table III.

The incidence of life-threatening toxicity was limited, with only one case of severe nephrotoxicity in arm B and almost negligible neurotoxicity.

Toxicity was, in general, more frequent in arm B. IFN, in addition to producing its typical toxicities (fever, asthenia, anorexia, flu-like syndrome), resulted in a worsening of chemotherapy side-effects (grade 3-4 vomiting and leucopenia 32% and 36% respectively). Carboplatin-

**Table I** Patient's characteristics

| | *MIP* (n = 23) | *MIP + IFN* (n = 27) | *CDDP + CBDCA* (n = 43) |
|---|---|---|---|
| | *Per cent of patients* | | |
| Stage | | | |
| III B | 34.8 | 11.1 | 18.6 |
| IV | 65.2 | 88.9 | 81.4 |
| Age | | | |
| <60 | 47.8 | 44.4 | 34.9 |
| ≥60 | 52.2 | 55.6 | 65.1 |
| Histology | | | |
| Squamous | 47.8 | 29.6 | 41.9 |
| Adenocarcinoma | 30.4 | 51.9 | 48.8 |
| Large cell | 8.7 | 18.5 | 2.3 |
| PS | | | |
| <2 | 78.2 | 77.7 | 88.4 |
| ≥2 | 8.8 | 14.8 | 11.6 |
| Sex | | | |
| Male | 87.0 | 88.9 | 88.4 |
| Female | 13.0 | 11.1 | 11.6 |
| Weight loss | | | |
| ≥10% | 4.3 | 7.4 | 14.0 |
| <10% | 43.5 | 40.7 | 27.9 |
| No | 43.5 | 48.1 | 41.9 |

**Figure 1** Overall survival. Median time to survival = 183.2 days.

**Table III** Treatment toxicity[a]

| | *MIP* (n = 21) | | *MIP + IFN* (n = 25) | | *CDDP + CBDCA* (n = 40) | |
|---|---|---|---|---|---|---|
| *WHO grade* | *1-2* | *3-4* | *1-2* | *3-4* | *1-2* | *3-4* |
| Nausea/vomiting | 76.2 | 19.0 | 68.0 | 32.0 | 67.5 | 7.5 |
| Alopecia | 42.8 | 23.0 | 52.0 | 20.0 | 45.0 | – |
| Mucositis | 28.6 | – | 12.0 | – | 12.5 | – |
| Diarrhoea | 4.8 | – | 16.0 | – | 7.5 | – |
| Fever | 14.3 | – | 68.0 | 4.0 | 20.0 | 5.0 |
| Constitutional | – | – | 36.0 | 4.0 | 7.5 | – |
| Asthenia | 66.7 | 9.5 | 76.0 | 20.0 | 50.0 | 7.5 |
| Anorexia | 66.7 | 4.7 | 72.0 | 20.0 | 47.5 | 10.0 |
| Leucopenia | 47.6 | 9.5 | 20.0 | 36.0 | 17.5 | 10.0 |
| Thrombocytopenia | – | 4.8 | 24.0 | 12.0 | 25.0 | 10.0 |
| Anaemia | 19.0 | – | 36.0 | 8.0 | 22.5 | 7.5 |

[a]Worst toxicity observed per patient.

**Table II** Response to treatment

| | *MIP* (n = 23) | *MIP + IFN* (n = 27) | *CDDP + CBDCA* (n = 43) |
|---|---|---|---|
| | *Per cent of patients* | | |
| Complete response | – | 3.7 | 12.4 |
| Partial response | 8.8 | 3.7 | 11.6 |
| Stable disease | 21.8 | 37.0 | 30.2 |
| Progressive disease | 17.4 | 14.4 | 30.2 |
| Early progression of death | 26.0 | 18.5 | 11.7 |
| Inadequate follow-up | – | – | 4.7 |
| Protocol violation | 4.3 | 7.4 | 2.3 |
| Early interruption for toxicity | 4.3 | 3.7 | – |
| Inadequate radiological documentation of response | 17.5 | 7.5 | 4.7 |
| Treatment refusal | – | 3.7 | 2.3 |

cisplatinum was the best-tolerated treatment: among 40 evaluable patients, we observed only three cases of grade 3-4 vomiting, 10% grade 3-4 thrombocytopenia and evident alopecia was virtually absent.

### Discussion

The three platinum-based chemotherapy regimens under investigation displayed poor anti-tumour activity in this randomised phase II study. The low response rate achieved could not be attributetd to inadequacy of dose intensity or study quality. These new chemotherapy combinations do not seem to represent a step forward compared with standard chemotherapy regimens, since the study had a 90% power against the alternative hypothesis of an activity of 30% in metastatic NSCLC. Therefore, it does not seem justified to explore them further and launch randomised phase III studies comparing the outcome of patients treated with these novel combinations *vs* standard regimes.

The activity of MIP was lower than that reported in previous studies. In fact, the response rate of MIP in non-randomised phase II studies (Giron *et al.*, 1987; Cullen *et al.*, 1988; Currie *et al.*, 1990; Mariani *et al.*, 1991) was always reported to be greater than 30% (37-69%) whereas, in the present study, the anti-tumour activity was clearly lower. This discrepancy may arise for a number of reasons: the selection of patients with more advanced disease (higher proportion of patients with stage IV disease), the strict methodology used to assess response and the use of an 'intention to treat' analysis (unevaluable patients were included in the denominator for calculating major response rate). However, it is our opinion that MIP activity was overestimated in previous phase II studies, as is often the case for single-institution phase II compared with multicentre phase III studies (Einhorn *et al.*, 1986). This strengthens the validity of the randomised phase II design as a more reliable tool to screen the activity of novel treatments. There are many advantages with studies of this type. Firstly, randomisation ensures that patients are centrally registered before treatment starts. This is essential to check eligibility and to ensure that all patients enrolled are reported upon. Second, patient selection, response criteria, dose modifications and reporting procedures are homogeneous among the participating centres. Finally, the central review of radiological material allows the inter-observer variability in response assessment to be reduced.

The addition of IFN to MIP did not improve the response rate. This is in contrast to one of our previous randomised studies, which showed an increased response rate when IFN was added to PEC chemotherapy (8.7% *vs* 21.5%) (Ardizzoni *et al.*, 1993). Given the low activity of PEC in that study, we aimed at exploring the potentiating activity of IFN with a more recent, and presumably more active, regimen. However, the results of the present study would not suggest a potentiating effect of IFN on MIP chemotherapy. This might be because of lower dose of IFN used in this study (3 MU compared with 5 MU in the previous study).

CDDP and CBDCA possess similar single-agent activity in advanced NSCLC (Bonomi *et al.*, 1989) with different dose-limiting toxicities. Although they share a common active metabolite and therefore a common mechanism of anti-tumour action, they have distinct pharmacodynamics and additive cytotoxicity in lung cancer cell lines (Hong *et al.*, 1985; Roed *et al.*, 1988; Cohen *et al.*, 1989). Therefore, in theory, the combination of CDDP and CBDCA might enable the delivery of a high total dose of 'platinum' and an enhanced tumour cell kill, without producing excessive addictive toxicity. The activity of this combination in our study is similar to that of other CDDP-containing regimens. Interestingly, toxicity was unexpectedly mild, with only 10% of the patients having grade 3-4 leucopenia or thrombocytopenia and virtually no case of complete alopecia. Our results are similar to those of two other studies. A CALGB phase II trial reported a response rate of 13% among 76 extensive NSCLC patients (Kreisman *et al.*, 1990). Despite the use of slightly higher chemotherapy doses than in the American study, we observed a lower incidence of severe haematological toxicity. The European Lung Cancer Working Party has recently published the results of a phase II randomised study of single-agent high-dose CDDP and moderate-dose CDDP combined with CBDCA. Although 50% less CBDCA was used in comparison with our study, they obtained a slightly better response rate (21%) with superimposable toxicity among 53 eligible patients (Sculier *et al.*, 1994). Therefore, CBDCA dose does not seem to play a major role in the activity and toxicity of the CDDP-CBDCA regimen.

In conclusion, none of the novel chemotherapy combinations under investigation in the present randomised phase II study appears to offer a significant therapeutic advantage over standard chemotherapy programmes. The combination of CBDCA and CDDP, given the low haematological and non-haematological toxicity, can be considered as an alternative to standard platinum-containing regimens. Phase II randomised trials are a reliable and quick method of screening and verifying the anti-tumour activity of novel agents or combinations which may better orient the design of comparative phase III randomised studies in NSCLC.

**Acknowledgements**
We thank Monica Guelfi and Simona Pastorino for data management and Justin Rainey for English review.

### References

ARDIZZONI A, SALVATI F, ROSSO R, BRUZZI P, RUBAGOTTI A, PENNUCCI MC, MARIANI GL, DE MARINIS F, PALLOTTA G, ANTILLI A, CRUCIANI AR, RINALDI M, TONACHELLA R, FIORETTI M, BARBERA S, MANTELLINI E, SORESI E, PASTORINO G, BELLI M, FERRARA G, VENTURINI M, SCAGLIOTTI G AND SANTI L FOR THE ITALIAN LUNG CANCER TASK FORCE (FONICAP). (1993). Combination of chemotherapy and recombinant alpha-interferon in advanced non-small cell lung cancer. Multicentric randomized FONICAP trial report. *Cancer*, **72**, 2929-2935.

BONOMI PD, FINKELSTEIN DM, RUCKDESCHEL JC, BLUM RH, GREEN MD, MASON B, HAHN R, TORMEY DC, HARRIS J, COMIS R AND GLICK J. (1989). Combination chemotherapy versus single agents followed by combination chemotherapy in stage IV non-small-cell lung cancer. A study of the Eastern Co-operative Oncology Group. *J. Clin. Oncol.*, **7**, 1602-1613.

COHEN JD, ROBINS HI AND SCHMITT CL. (1989). Tumoricidal interaction of hyperthermia with carboplatin, cisplatin and etoposide. *Cancer Lett.*, **44**, 205-210.

CULLEN MH, JOSHI R, CHETIYAWARDANA AD AND WOODROFFE CM. (1988). Mitomycin, ifosfamide and cisplatin in non-small cell lung cancer: treatment good enough to compare. *Br. J. Cancer*, **58**, 359-361.

CURRIE DC, MILES DW, DRAKE JS, RUDD R, SPIRO SG, EARL HM, HARDER PG, TOBIAS JS AND SOUHAMI RL. (1990). Mitomycin, ifosfamide and cisplatin in non-small cell lung cancer. *Cancer Chemother. Pharmacol.*, **25**, 380-381.

EINHORN LH, LOEHRER PJ, WILLIAMS SD, MEYERS S, GABRYS T, NATTAN SR, WOODBURN R, DRASGA R, SONGER J, FISHER W, STEPHENS D AND HUI S. (1986). Random prospective study of vindesine versus vindesine plus high-dose cisplatin versus vindesine plus cisplatin plus mitomycin C in advanced non-small cell lung cancer. *J. Clin. Oncol.*, **4**, 1037-1043.

GIRON G, ORDONEZ A, JALON JI AND BARON MG. (1987). Combination chemotherapy with ifosfamide, mitomycin and cisplatin in advanced non-small cell lung cancer. *Cancer Treat. Rep.*, **71**, 851-853.

HONG R, OHNUMA T, INOVE S, SUN AS AND HOLLAND JF. (1985). Cisplatin and carboplatin in combination. *Proc. Am. Assoc. Cancer Res.*, **26**, 260.

HRYNIUK, W. & BUSH, H. (1984). The importance of dose intensity in chemotherapy of metastatic breast cancer. *J. Clin. Oncol.*, **2**, 1281-1288.

IHDE DC. (1992). Chemotherapy of lung cancer. *N. Engl. J. Med.*, **327**, 1434-1441.

KREISMAN H, GOUTSOU M, MODEAS C, GRAZIANO SL, COSTANZA ME AND GREEN MR. (1990). Cisplatin-carboplatin therapy in extensive non-small cell lung cancer: a Cancer & Leukemia Group B study. *Eur. J. Cancer*, **26**, 1057-1060.

MARIANI GL, PENNUCCI MC, ADDAMO GF, VENTURINI M, ARDIZZONI A AND ROSSO R. (1991). A pilot study of mitomycin, ifosfamide and cisplatin as outpatient combination chemotherapy for advanced non-small cell lung cancer. *Tumori*, **77**, 511-513.

PICCART MJ, NOGARET JM, MARCELIS L, LONGREE H, RIES F, KAINS JP, GOBERT P, DOMANGE AM, SCULIER JP, GOMPEL C AND THE BELGIAN STUDY GROUP FOR OVARIAN CARCINOMA. (1990). Cisplatin combined with carboplatin: a new way of intensification of platinum dose in the treatment of advanced ovarian cancer. *J. Natl Cancer Inst.*, **82**, 703-707.

ROED H, VINDELOV LL, CHRISTENSEN IJ, SPANG-THOMSEN M AND HANSEN HH. (1988). The cytostatic activity of cisplatin, carboplatin and teniposide alone and combined determined on four human small cell lung cancer cell lines by clonogenic assay. *Eur. J. Cancer Clin. Oncol.*, **24**, 247-253.

SCULIER JP, KLASTERSKY J, GINER V, BUREAU G, THIRIAUX J, DABOUIS G, EFREMIDIS A, RIES F, BERCHIER MC, SERGYSELS R, MOMMEN P AND PAESMANS M FOR THE EUROPEAN LUNG CANCER WORKING PARTY. (1994). Phase II randomized trial comparing high-dose cisplatin with moderate-dose cisplatin and carboplatin in patients with advanced non-small cell lung cancer. *J. Clin. Oncol.*, **12**, 353-359.

SIMON R. (1989). Optimal two stage design for phase II clinical trials. *Controlled Clin. Trials*, **10**, 1-10.